# Bilingual Families Align Their Languages During Naturalistic Interactions: Evidence from Two Bilingual Communities

**DOI:** 10.3390/bs16050788

**Published:** 2026-05-15

**Authors:** Laia Fibla, Jessica E. Kosie, Rachel Ka-Ying Tsui, Christine E. Potter, Casey Lew-Williams, Krista Byers-Heinlein

**Affiliations:** 1 Department of Psychology, Concordia University, Montreal, QC H4B 1R6, Canada; kaying.tsui@riken.jp (R.K.-Y.T.); k.byers@concordia.ca (K.B.-H.); 2 Department of Psychology, Princeton University, Princeton, NJ 08544, USA; jessica.kosie@asu.edu (J.E.K.); cepotter2@utep.edu (C.E.P.); caseylw@princeton.edu (C.L.-W.); 3 School of Social and Behavioral Sciences, Arizona State University, Phoenix, AZ 85069, USA; 4 RIKEN Center for Brain Science, Wako 351-0198, Saitama, Japan; 5 Department of Psychology, University of Texas at El Paso, El Paso, TX 79968, USA

**Keywords:** bilingual language acquisition, language alignment, conversational-turns, caregiver–child interaction, family language use, cross-community, cross-cultural

## Abstract

Bilingual children learn their languages through rich interactions with caregivers within dynamic family contexts. However, little is known about how families align their two languages to support bilingual acquisition and how this varies across bilingual communities. This study examines language choice alignment across two communities: French–English families in Quebec (Canada) and Spanish–English families in New Jersey (United States). Thirty-nine children aged 18–35 months and their families were video-recorded during two 20 min home play sessions—one with a primary caregiver only and one including additional household members. Utterances were coded for speaker identity and language. We found strong turn-by-turn alignment between primary caregivers and children across both communities and sessions, with observed alignment exceeding chance in ⅔ of analyzed sessions at rates 20–22% above baseline. Other family members showed weaker correspondence with children’s language choices. Children’s alignment was modulated by language exposure and age, whereas caregivers’ alignment only decreased when additional members were present. These findings demonstrate that primary caregivers and their bilingual children align language choices consistently across diverse family configurations and communities. This linguistic coupling may support bilingual development across diverse interaction contexts, highlighting primary caregivers’ central role in early bilingual experiences across societies where bilingualism is and is not the norm.

## 1. Bilingual Families Align Their Languages During Naturalistic Interactions: Evidence from Two Bilingual Communities

Bilingual families navigate complex decisions about language use in their daily interactions. A mother might switch from French to English when helping her toddler understand a new word, or a child might respond in Spanish when their parent addresses them in English. These moment-to-moment language choices create dynamic patterns of coordination—or misalignment—between family members. This study examines language alignment, defined as the degree to which caregivers and children match their language use during naturalistic interactions, across two bilingual communities with different sociolinguistic contexts: French–English families in Quebec, Canada, and Spanish–English families in New Jersey, United States. In the context of the Systems Framework of Bilingual Development (Byers-Heinlein et al., 2025; Castellana & Benitez, 2026), we investigate how this bidirectional coordination process varies across different configurations of family members and community contexts, and what factors predict alignment strength.

Understanding language alignment is crucial because bilingual development emerges from dynamic interactions across multiple levels of the environment (Byers-Heinlein et al., 2025). At the individual level, children bring cognitive capacities and language-learning abilities that shape how they process and respond to multilingual input (Byers-Heinlein & Fennell, 2014; Werker & Byers-Heinlein, 2008). At the interpersonal level, families create immediate language environments through their daily language choices (De Houwer, 2011), interaction patterns (Hoff, 2006; Carbajal & Peperkamp, 2020), household configurations (Shneidman & Goldin-Meadow, 2012; Ramírez-Esparza et al., 2017), and caregiving arrangements (Sander-Montant et al., 2025; Orena et al., 2019). Finally, at the societal level, broader contexts determine the support for different languages (De Houwer, 2015; Cychosz et al., 2025). Most research has focused on unidirectional influences, for example, how caregivers shape children’s language environments (e.g., Marchman et al., 2017; Orena et al., 2020), while overlooking the bidirectional, dynamic processes through which families coordinate their language use in real-time. Moreover, the vast majority of studies on bilingual acquisition have focused on a single bilingual context, limiting our ability to distinguish general alignment patterns from community-specific factors (Poplack, 1988; Kidd & Garcia, 2022).

Communication fundamentally involves coordination between partners. In monolingual contexts, extensive research documents how speakers naturally align their language use over time across phonological, syntactic, and semantic dimensions (Brennan & Hanna, 2009). During infancy, caregivers adapt the complexity of their speech to match their children’s developing abilities, supporting language acquisition through this responsive coordination (Snow, 1972; Yurovsky et al., 2016; Denby & Yurovsky, 2019). Bilingual interactions introduce an additional layer of complexity: speakers must coordinate not only how they speak, but which language they use. Highly proficient bilingual adults, adolescents and children typically adapt their language choices to their communication partners to facilitate understanding (Costa et al., 2006; Gampe et al., 2019), often based on perceived language competence (Gasiorek et al., 2022). Bilingual caregivers face unique challenges in this coordination process, as they must simultaneously consider their own language abilities, their children’s developing proficiency in each language, and their family’s language goals. These complex decisions can be understood through the lens of family language policy, which encompasses the language ideologies, management strategies, and actual language practices that families enact in the home (K. A. King et al., 2008; Spolsky, 2012). While family language policy research has documented how parental beliefs and deliberate planning shape children’s language environments (K. A. King & Fogle, 2013; Curdt-Christiansen, 2009), less attention has been paid to the real-time language practices through which these policies are enacted during moment-to-moment interaction. For example, parents may choose to maintain consistent language use to teach vocabulary in a specific language, or they may flexibly switch languages to ensure comprehension and maintain engagement (Kremin et al., 2022). Parents’ moment-to-moment language decisions are further complicated by factors such as parents’ own language dominance, their comfort level in each language, their beliefs about effective bilingual parenting strategies (K. King & Fogle, 2006), and their attitudes and concerns about childhood bilingualism (Kircher et al., 2022; Quirk et al., 2024). Family language use might also be determined by who is present during communicative exchanges, and thus, language dynamics might vary based on household configuration such as the number of adult family members and siblings (Hoff et al., 2014; Shneidman & Goldin-Meadow, 2012). The complexity of these choices means that families’ alignment patterns may vary considerably over time and between one another.

When caregivers use different languages from one turn to the next, these misalignments can also be understood as code-switches—alternations between languages within or across conversational turns. Code-switching is a natural and common feature of bilingual interaction (Grosjean, 2010; Poplack, 1988), and caregivers may switch languages during interactions with their children for various reasons. Research documenting parental language switching shows that the amount and type of code-switching—both within and across speakers—varies as a function of caregivers’ level of language expertise, family language strategies, cultural norms, and for pedagogical purposes such as teaching new words or boosting comprehension (Byers-Heinlein, 2013; Kremin et al., 2022; Paquette & Byers-Heinlein, 2025). Further, at the broader contextual level, some communities embrace fluid language mixing as a natural part of bilingual interaction, while others maintain stronger preferences for language separation (Gardner-Chloros, 2009). These patterns highlight the complexity of language alignment in bilingual development, where misalignment may sometimes reflect strategic communication choices rather than coordination failures. Community norms around language separation versus mixing are often internalized by caregivers and shape their home language practices and ideologies (Curdt-Christiansen, 2009; De Houwer, 2015), influencing their general and moment-to-moment language choices even in private, dyadic interactions with their children.

Language alignment, however, depends not only on parents’ language choices, but also on children’s contribution to the interaction. Within the Systems Framework of Bilingual Development (Byers-Heinlein et al., 2025), children’s individual abilities interact with family language environments to shape coordination patterns. Bilingual children demonstrate early sensitivity to their communication partners’ language choices (Comeau et al., 2003; Paradis & Nicoladis, 2007) and can sometimes successfully adjust their own language use when interacting with adults (De Houwer, 1983; Deuchar & Quay, 2001; Genesee et al., 1996). However, multiple factors influence why children might speak a different language than their conversational partner, including their overall language expertise, proficiency in the target language, specific word knowledge, developing cognitive control abilities, or contextual cues such as consistent language use in particular settings or with particular individuals (Gross & Kaushanskaya, 2020; Gross et al., 2022; Hansen et al., 2025).

Only one previous study has specifically examined language alignment between young bilingual children and their caregivers. Slawny et al. (2025) found that 4–5-year-old Spanish–English children and their parents aligned their language choices during naturalistic play interactions, with language dominance influencing this coordination. However, several limitations remain. First, this study focused on a single caregiver and community context, limiting our understanding of how alignment varies across different family configurations and sociolinguistic environments. Second, like much prior work on bilingual interaction patterns, observed matching rates were not compared against chance expectations. When speakers have strong individual preferences for one language (e.g., predominantly using English), they may appear to match each other simply because both frequently use the same language, rather than because they are actively coordinating. To establish whether interlocutors are engaging in in-the-moment alignment, it is necessary to demonstrate that sequential dependencies between speakers’ language choices exceed what would be expected if each speaker independently selected languages based solely on their individual preferences.

This study addresses key gaps in our understanding of language alignment by examining coordination patterns across multiple configurations of household members and community contexts. We video recorded thirty-nine children aged 18-to-35 months and their bilingual families during two naturalistic home interactions. This age range captures a critical period in bilingual development when children are rapidly expanding their vocabularies and beginning to produce multi-word utterances (Fenson et al., 1994), yet may still have limited cognitive control abilities that could affect their language coordination skills (Bialystok, 2011). The first session involved one-on-one play between the child and a primary caregiver, while the second session included additional household members such as other parents, siblings, and grandparents, which allowed us to observe language alignment with different caregivers and in multi-speaker contexts. Twenty families were French–English bilinguals from Quebec, Canada, where both languages enjoy official status and institutional support, with widespread bilingual education and positive societal attitudes toward bilingualism (Kircher et al., 2022). The remaining nineteen families were Spanish–English bilinguals from New Jersey, United States, where Spanish functions as a heritage language with limited official recognition despite being widely spoken in many communities (Anderson & Toribio, 2007; Montanari et al., 2019). Research suggests that these two bilingual communities show different rates of code-switching, with Spanish–English bilingual parents generally switching more often (Bail et al., 2015) than French–English bilinguals (Paquette & Byers-Heinlein, 2025). Importantly, both communities also show different within-speaker and across-speaker switching patterns when different family members are present during interactions: Spanish–English bilingual families show similar rates during one-to-one interactions and multi-party interactions, but French–English bilinguals show considerably more across-speaker switches when multiple speakers are present (Kosie et al., 2026). These differences in switching patterns might reflect social norms from each community’s sociolinguistic context, which may create distinct pressures and opportunities that influence how families coordinate their language choices during interactions (Anderson & Toribio, 2007; Tulloch & Hoff, 2023; Kircher et al., 2022; Kremin et al., 2022). The present study applies a comparative cross-community lens to examine language alignment in bilingual families, extending an approach that has proven fruitful in adult bilingual speech research (Balam et al., 2020; Couto et al., 2021). This design allows us not only to examine how different sociolinguistic contexts and community norms might influence family language alignment patterns, but also to identify which patterns generalize across communities—a distinction that single-community studies cannot make.

We examined alignment behaviors across different communities and interpersonal contexts using three metrics: overall, bidirectional, and moment-to-moment alignment across language turns (compared to chance using Monte Carlo simulations). This approach allowed us to investigate three primary research questions: (RQ1) Are caregivers and children aligned in their language choices during interactions, and how does this vary across caregivers and interpersonal contexts (one caregiver versus multiple caregivers)? (RQ2) Do alignment patterns differ between families in Quebec (French–English) and New Jersey (Spanish–English), reflecting different sociolinguistic contexts? (RQ3) What individual factors predict alignment strength, including child age, language dominance and caregiver characteristics?

Based on the Systems Framework of Bilingual Development (Byers-Heinlein et al., 2025) and existing literature, we made several predictions. First, we expected that children would be more aligned with their primary caregivers than with other family members, and that their alignment with their primary caregiver would be stronger during one-on-one interactions compared to when additional family members are present. This would reflect primary caregivers’ greater language input provision and responsiveness to children’s needs, as well as the reduced complexity of dyadic versus group interactions. Second, we anticipated that French–English families in Quebec would show different alignment patterns than Spanish–English families in New Jersey, reflecting the contrasting language status and community support in these contexts, although we did not have any specific or directional predictions. Third, we expected that child factors such as age and language dominance, along with family factors such as caregiver language proficiency, would systematically predict alignment strength, with more balanced bilingual exposure and higher caregiver proficiency supporting closer alignment.

## 2. Method

### 2.1. Participants

Our final sample included 39 families with children aged 18 to 35 months (*M* = 24.76, *SD* = 5.47; 24 girls, 15 boys). Two families from the original recruited sample of 41 were excluded: one because the child was regularly exposed to a third language for more than 10% of the time, and one during data cleaning because none of the child’s speech could be identified. Of the 39 remaining families, 28 participated in both sessions; the remaining 11 completed Session A only and are excluded from analyses comparing the two sessions. Dropout was unequally distributed across communities: 4 French–English families and 7 Spanish–English families completed Session A only. For families who completed both sessions, the same primary caregiver was present in both recordings, with one or more additional household members present during Session B. Two families did not return questionnaire data (both from the Spanish–English community) and are therefore excluded from analyses examining caregiver language ability and child language exposure.

Families were recruited using targeted Facebook ads and via existing laboratory databases of families interested in participating in research. Ethics approval was obtained from the Concordia University Human Research Ethics Board (Certification numbers: 10000439, UH211-041-1) and from the Princeton University Institutional Review Board (Protocol Number: 7117). Families were informed of the study and gave consent to participate prior to the data collection. All children in the study were exposed to English and French or Spanish for at least 10% of their daily language input, and had no diagnosed developmental delays or hearing impairments. Data collection was completed between August 2020 and October 2020 in Quebec, and July 2020 through March 2021 in New Jersey, which was during the COVID-19 pandemic lockdowns. The sample included three sets of twins (2 from the English–Spanish community and 1 from the French–English community). In two cases, both twins were tested in the same session but only alignment with the twin that had more speech overall was included in the analysis. Thus, in those sessions one of the twins was considered the target child, whereas the other was considered a sibling (for Session A speech from the sibling twin was excluded from analysis). For the last twin set, each twin completed Session A with a different caregiver, and both were present during Session B. For that set, we included both Session A, but Session B only counted for one of the twins.

#### 2.1.1. French–English Sample (Quebec, Canada)

In the French–English subsample, children’s lifetime exposure to English ranged from 10% to 70% (*M* = 46.70; *SD* = 17.02) and their exposure to French ranged from 25% to 90% (*M* = 52.30; *SD* = 17.73). Parents reported that 55% of children knew more English words, and 45% knew more French words. Most caregivers in the sample had completed higher education: Master’s Degree 25%, Bachelor’s Degree 50%, College Certificate/Diploma 10%, Associate’s Degree 5%, Some College 10%.

During Session A (*n* = 20 families), all children in the French–English subsample interacted with their mother. In Session B (*n* = 16 families) the same caregiver from Session A and additional household members were present, including: 16 mothers, 16 fathers, 2 siblings, and 3 grandparents.

#### 2.1.2. Spanish–English Sample (New Jersey, United States)

In the Spanish–English subsample, children’s exposure to English ranged from 24.50% to 85% (*M* = 54.29; *SD* = 17.06) and their exposure to Spanish ranged from 15% to 74.50% (*M* = 45.18; *SD* = 17.07). Parents reported that 53% of children knew more English words and 37% knew more Spanish words. Most caregivers in the sample had completed higher education: PhD/Doctoral Degree 16%, Master’s Degree 37%, Bachelor’s Degree 16%, Associate’s Degree 16%, Some College 5%.

During Session A (*n* = 19 families), 15 children in the Spanish–English subsample interacted with their mother and 4 with their father. During Session B (*n* = 12 families) the same caregiver from Session A and additional household members were present, including: 12 mothers, 4 fathers, 6 siblings, 3 grandparents, and 1 nanny. Note that the sample size for this group was reduced by two participants due to missing questionnaire data in the analysis looking at caregiver language ability and child language exposure (Session A *n* = 17; Session B *n* = 10).

### 2.2. Materials

Prior to the free-play session, participants were emailed an online questionnaire that gathered demographic and health information of the child (e.g., race, ethnicity, number of siblings and household members, health and birth history), as well as estimates of current and cumulative language exposure to English, French and/or Spanish and the language ability for each of the child’s regular caregivers (e.g., age of acquisition, regular use, general ability, use with the child). We also gathered socio-economic information from caregivers such as their level of education; income data were not collected due to cultural sensitivity concerns across sites. Caregivers self-rated their own language abilities using a 1–7-point Likert scale, where 1 indicated knowing a few words in a language and 7 indicated being a native speaker of that language. The same questionnaire was used across sites, and it was available in English, French, and Spanish. The questionnaire included an open-ended question where caregivers could add information about language use and language switching practices in the family. All our materials can be freely accessed (Fibla et al., 2026, April 1).

### 2.3. Measures

Several variables derived from the parental questionnaire were used as predictors in subsequent analyses.

*Child language exposure.* Children’s proportion of exposure to each language was calculated from parental reports using an online questionnaire. The language exposure section was a shortened adaptation of the MAPLE (Multilingual Approaches to Parent Language Exposure; Byers-Heinlein et al., 2020), in which parents reported the percentage of words spoken directly to their child in each language on a typical day and across their child’s entire lifetime. The lifetime exposure estimate was used as a predictor in subsequent analyses and as a proxy for children’s relative language ability, given the strong relationship between language exposure and vocabulary (Byers-Heinlein et al., 2024; Thordardottir, 2011; Place & Hoff, 2011). Studies of bilingual children vary in terms of the minimum threshold of second language exposure they use to define bilingualism (Rocha-Hidalgo & Barr, 2023), and in the current research, children were included if they received at least 10% lifetime exposure to each language. We adopted this broad criterion to maximize sample diversity, consistent with approaches that treat bilingual exposure as a continuous dimension rather than a categorical cutoff (Kremin & Byers-Heinlein, 2021). The mean exposure in both communities was approximately 50%, indicating that most children were fairly balanced.

*Child age.* Child age in months at the time of recording was used as a continuous predictor.

*Caregiver language ability.* Primary caregivers’ self-rated proficiency in each language was assessed using 7-point Likert scales and measured across four different questions related to age of acquisition, daily use, regular use with the child, and self-rated proficiency. A factor analysis with promax rotation revealed a two-factor solution (KMO = 0.80; Bartlett’s test *p* < .001). Factor 1 captured non-English language use and proficiency (loadings: 0.68–0.94), while Factor 2 reflected English language use and proficiency (loadings: 0.50–1.08), with both factors showing negative loadings for age of acquisition. The two factors were moderately negatively correlated (*r* = −0.53). Based on these results, we selected self-rated language proficiency in each language as our measure of caregiver language ability. Average language ability in French–English caregivers was 6.85 for English (*SD* = 0.49; range = 5–7) and 6.30 for French (*SD* = 1.17; range = 3–7). Average language ability for Spanish–English caregivers was 6.65 for English (*SD* = 0.79; range = 4–7) and 5.94 for Spanish (*SD* = 1.75; range = 1–7).

All continuous predictors were scaled and centered prior to inclusion in statistical models.

### 2.4. Procedure

Families were video-recorded over Zoom at home during two 20 min free play sessions. Families were instructed to play as they normally would, using toys and materials available in their home environment. In Session A, infants played with only one primary caregiver. In Session B, one or more additional household members were present, determined by each family’s availability and preference. The two sessions occurred approximately 10 days apart (*M* = 9.71; range = 0–36).

### 2.5. Video Coding

For the full 20 min session, we coded onsets and offsets of all utterances and tagged each for speaker identity and, where possible, language(s). When an utterance included both languages, we segmented it by language and tagged each segment separately, allowing us to calculate what portion of the utterance was spoken in each language. Coding was completed by a team of 8 native speakers of English, French and Spanish (5 in Canada and 3 in the US), who hand-coded and transcribed the play sessions using the ELAN annotation software versions 6.3 and 6.4 (Wittenburg et al., 2006) using adapted protocols based on a coding scheme developed by the Analyzing Child Language Around the World (ACLEW) group (Soderstrom et al., 2021; adapted coding protocols can be accessed on our OSF repository). Our adaptation of the ACLEW coding scheme allowed us to more easily calculate alignment and switching because we added a separate coding tier to explicitly annotate the language of each utterance. We created careful protocols for decision-making during ambiguous cases and held regular meetings with the coding teams to discuss discrepancies.

In addition, prior to coding the data, coders underwent two levels of training. First, they completed the training designed by ACLEW (Casillas et al., 2023, August 26), a validated community standard for coding speech from naturalistic data, that covers both the basics of ELAN and the standard transcription conventions, and includes a gold standard certification test requiring coders to reach 85% accuracy on all annotation subparts and an overall score of 95% or higher to pass. Next, coders annotated a 10 min training file integrating our study-specific adaptations for bilingual data, which was reviewed by an experienced coder who provided feedback when coding differed from our protocols. Based on performance on both the ACLEW gold standard test and our study-specific training file, one of the authors gave approval for coders to begin working on the study data. When performance did not meet the required threshold, training was repeated until the criteria were met. After completing this training, coders were considered reliable and started working on the study data. Disagreements and coding questions that arose during the coding process were discussed at bi-weekly team meetings, and decisions were recorded in a shared annotated logbook accessible to coders across both sites to ensure consistency throughout the coding process.

From the coding, we computed language use proportions for each individual speaker by dividing the time they spent speaking each language (English, French/Spanish) by their total speech time. These proportions were calculated separately for caregivers and children within each session. In addition, we identified conversational turns as instances where a household member’s utterance was followed by the child’s utterance, or vice versa. We tagged these turns between the child and any household member and labeled them as a match if the language used across speakers was the same or as a switch if the language changed.

## 3. Results

### 3.1. Analytic Overview

We conducted three complementary analyses to address our research questions, examining language alignment at progressively finer timescales. Analysis 1 tested whether caregivers and children showed correspondence in terms of the overall proportion in which they used each language. Analysis 2 examined bidirectional influence, testing whether the language used by each speaker predicted the language used by other speakers in the household. Analysis 3 examined sequential alignment, by testing whether speakers matched language choices turn-by-turn above chance expectations. Across all analyses, we compared primary caregivers versus other family members and Session A (one-on-one) versus Session B (multiple household members) to address how alignment varies by caregiver type and interpersonal context (RQ1). We tested for differences between French–English and Spanish–English communities throughout (RQ2). Individual predictors including child age, language exposure, and caregiver language ability were examined in Analyses 2 and 3 (RQ3). Where multiple observations per family were available, as in the turn-by-turn alignment models (Analysis 3), we included random effects for family to account for within-family variability. In proportion-based models (Analyses 1 and 2), each family contributed a single observation per session and community combination, precluding the inclusion of random effects.

### 3.2. Preliminary Analyses: Overall Language Use Patterns

We first set out to describe overall patterns of language use to characterize the linguistic environments in which alignment occurred. Across both communities, primary caregivers and children showed substantial variability in their percentage of use of English versus French/Spanish, reflecting diverse family language practices. Overall, participants used each language in similar proportions across sites: French–English bilinguals used English 42% of the time and French 58% of the time, while Spanish–English bilinguals used English 58% of the time and Spanish 42% of the time.

Table 1 presents the percentage of English use by session, bilingual community, and speaker. Both communities showed high variability in language mixing, with individual participants ranging from exclusive use of one language (0% or 100% English) to balanced bilingual usage. Session differences emerged in both communities but in opposite directions. French–English children used less English overall in Session B (38.16%) than in Session A (49.42%), whereas Spanish–English children used more English in Session B (60.80%) than in Session A (55.36%).

In some cases, the language of an utterance could not be determined, and these were excluded from analyses. In French–English families, primary caregivers spoke in an identifiable language 93.39% of the time (*SD* = 5.28, range = 82–100%), other household members 89.88% of the time (*SD* = 7.95, range = 68–99%), and children 43.77% of the time (*SD* = 29.33, range = 0–97%). In Spanish–English families, primary caregivers spoke in an identifiable language 91.66% of the time (*SD* = 7.99, range = 65–100%), other household members 82.32% of the time (*SD* = 21.11, range = 37–99%), and children 46.90% of the time (*SD* = 26.84, range = 2–91%). We examined whether the proportion of child speech tagged as unknown decreased with age. A generalized linear model with a quasibinomial distribution and logit link (to account for overdispersion) predicted the proportion of unknown speech from child age, bilingual community, and session, including all interactions. The model structure in R syntax was:

glm(ProportionUnknownLang~ChildAge * Session * Community)

The model revealed a significant negative effect of age (*OR* = 0.33, 95% CI [0.16, 0.58], *t* = −3.51, *p* < .001), indicating that older children produced less speech where the language was unidentifiable. Specifically, for each additional month of age, the proportion of speech of unknown language decreased. No significant effects emerged for community (*OR* = 1.04, 95% CI [0.52, 2.09], *t* = 0.11, *p* = .915), session (*OR* = 0.99, 95% CI [0.49, 1.99], *t* = −0.03, *p* = .974), or any of the interactions (all *p*s > 0.266).

### 3.3. Analysis 1. Correspondence in Language Choices Between Caregivers and Children

To examine whether caregivers and children were aligned in their overall language choices, we calculated correlations between the proportion of English speech produced by each speaker and the target child. These analyses tested whether speakers who used more English had children who also used more English, indicating correspondence in language selection at the level of overall proportions. Analysis 1 included all 39 families for Session A (French–English: *n* = 20; Spanish–English: *n* = 19) and 28 families for Session B (French–English: *n* = 16; Spanish–English: *n* = 12).

**Figure 1:**
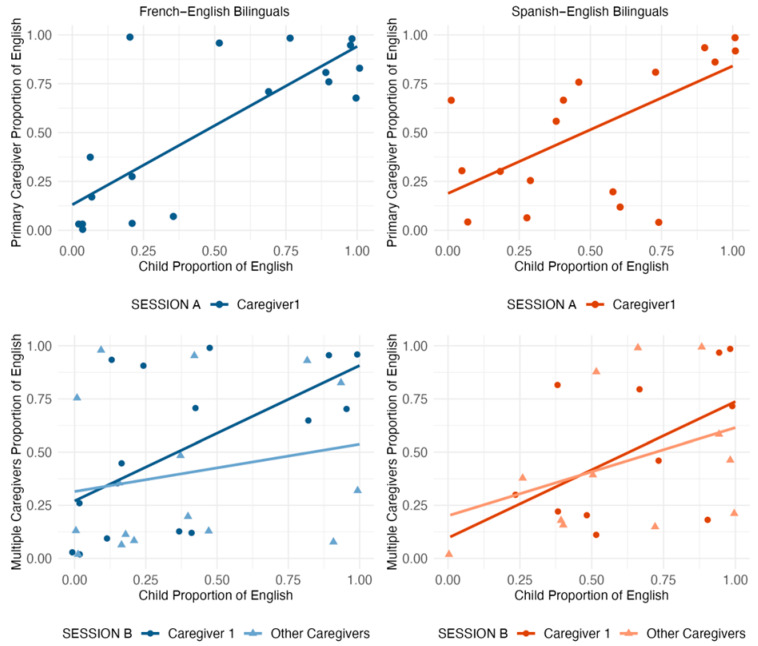
Proportion of English used by a primary caregiver (Caregiver 1) and the child in Session A (**top panels**), and split by all other household members (Other Caregivers) in Session B (**bottom panels**). Individual dots plot data from individual caregiver-infant matching.

Primary caregivers and their children were strongly aligned in their language choices across both communities regardless of whether they interacted one-on-one or with other family members present. In Session A during one-on-one interactions, correlations were strong amongst both French–English (*r*(18) = 0.81, *p* < .001) and Spanish–English (*r*(17) = 0.64, *p* = .003) families. To test whether these correlations differed across communities, we used Fisher’s r-to-z transformation test for independent groups (Fisher, 1925). The correlation between children’s and primary caregivers’ language choices in Session A did not differ significantly across bilingual communities (*z* = 1.05, *p* = .294, 95% CI for r difference [−0.15, 0.56]). This pattern of alignment between children and their primary caregivers persisted in Session B when additional family members were present, with significant correlations for French–English (*r*(14) = 0.60, *p* = .014) families and for Spanish–English (*r*(10) = 0.60, *p* = .039) families. The correlation between children’s and primary caregivers’ language choices in Session B did not differ significantly across bilingual communities (*z* = 0.01, *p* = .995, 95% CI for r difference [−0.53, 0.61]). Thus, primary caregivers and children used each language in similar proportions regardless of whether other family members were present (see Figure 1).

In contrast to primary caregivers, other family members in Session B showed weaker correspondence with children’s language choices, which indicates that other family members’ language choices were less closely aligned with children’s patterns than were primary caregivers’ choices. The correlations between children’s and other family members’ language choices were not significant in French–English families (*r*(14) = 0.21, *p* = .432) nor in Spanish–English families (*r*(10) = 0.39, *p* = .211). This weaker alignment could reflect that other family members vary more in their language proficiency and may be less attuned to children’s moment-to-moment language choices than primary caregivers. To measure whether these correlations were significantly weaker than those observed with primary caregivers, we used Steiger’s (1980) test for the difference between two overlapping dependent correlations. The difference was not significant in either community (French–English: *z* = 1.02, *p* = .308; Spanish–English: *z* = 0.58, *p* = .565), suggesting that while other family members showed numerically weaker alignment with children’s language choices than primary caregivers, the study was likely underpowered to detect this difference given the small subsample sizes (*n* = 16 and *n* = 12 respectively).

### 3.4. Analysis 2: Bidirectional Influences on Language Use

The strong correlations demonstrate correspondence in language choices, but do not reveal the directionality of influence. To test whether this pattern reflects bidirectional adaptation, we conducted three generalized linear models with quasibinomial distribution and logit link, examining: (1) whether children’s overall use of a particular language predicted caregivers’ overall use of that language, (2) whether caregivers’ overall use of a language predicted children’s overall use of that language, and (3) whether child use of a language predicted other family members’ use of that language. All models included session (Session A vs. Session B) and community (Canada vs. US) as factors. Individual predictors (child age, language exposure, caregiver language ability) were tested as covariates and retained only when they significantly improved model fit. Analysis 2 included 36 families for Session A (French–English: *n* = 20; Spanish–English: *n* = 17) and 26 families for Session B (French–English: *n* = 16; Spanish–English: *n* = 10), with two Spanish–English families excluded due to missing questionnaire data. Note that the third model with other family members’ language use did not include session as a factor since that model included only data from Session B.

#### 3.4.1. Primary Caregiver Language Use Model

We investigated whether the primary caregivers’ use of a language was predicted by children’s use of that language across sessions and bilingual communities. Since caregivers’ comfort and proficiency in a language may influence how much they use it, we tested whether including caregiver language ability improved our caregiver model. Model comparisons revealed that including caregiver language ability as a predictor significantly improved model fit, *F*(1, 117) = 6.80, *p* = .010, but not when entered as an interacting term, *F*(7, 110) = 0.41, *p* = .897. Therefore, the most simple model was used. The final model in R syntax was:

glm(ProportionCaregiverSpeaking~ProportionChildSpeaking * Session * Community + CaregiverLangAbility)

The model revealed a significant positive effect of child speech proportion (*OR* = 62.00, 95% CI [17.19, 270.48], *t* = 5.91, *p* < .001), indicating that caregivers spoke more when children spoke more. Caregiver language ability also predicted their language choices (*OR* = 1.46, 95% CI [1.09, 1.98], *t* = 2.49, *p* = .013), with higher ability in a language associated with greater use of that language. No significant main effects were found for session (*OR* = 2.23, 95% CI [0.70, 7.27], *t* = 1.35, *p* = .176) or bilingual community (*OR* = 1.53, 95% CI [0.43, 5.40], *t* = 0.67, *p* = .505), nor for the remaining interactions (all *p*s > 0.097). The relationship between child and caregiver use of a particular language was consistent across sessions and communities (*OR* = 1.28, 95% CI [0.06, 29.75], *t* = 0.16, *p* = .875). The model accounted for substantial variance, reducing the null deviance from 84.88 to a residual deviance of 45.75 (117 df).

#### 3.4.2. Child Language Use Model

We investigated whether children’s use of a particular language was predicted by the primary caregiver’s use of that language, session and bilingual community. A model including child language exposure as a main effect provided significantly better fit than a model without it, *F*(1, 117) = 14.52, *p* < .001, but including it as an interacting term did not significantly improve model fit, *F*(7, 110) = 0.26, *p* = .966. Thus, the simpler model was selected. We also tested if adding the child’s age improved the model, but it did not significantly improve model fit, *F*(1, 116) = 0.00, *p* = .980, and therefore, it was removed. The final model in R syntax was:

glm(ProportionChildSpeaking~ProportionCaregiverSpeaking * Session * Community + ChildLangExposure)

The model revealed a significant positive effect of caregiver speech proportion (*OR* = 19.05, 95% CI [5.00, 84.10], *t* = 4.12, *p* < .001), indicating that children spoke more when caregivers spoke more. Child language exposure also showed a significant positive effect (*OR* = 2.02, 95% CI [1.40, 2.96], *t* = 3.68, *p* < .001), such that children with greater language exposure spoke proportionally more in that language during the sessions. A marginally significant interaction between caregiver speech proportion and session (*OR* = 0.16, 95% CI [0.02, 1.04], *t* = −1.91, *p* = .056) suggested a trend toward weaker caregiver–child matching in Session B compared to Session A. No significant main effects were found for community (*OR* = 1.79, 95% CI [0.56, 5.77], *t* = 0.99, *p* = .323) or session (*OR* = 2.47, 95% CI [0.79, 7.93], *t* = 1.55, *p* = .122), nor for the remaining interactions (all *p*s > 0.214). The model accounted for substantial variance, reducing the null deviance from 84.98 to a residual deviance of 44.87 (117 df).

#### 3.4.3. Other Household Members’ Language Use Models

We examined whether other household members’ use of a particular language in Session B was predicted by children’s use of that language, by community, and by their interaction. In cases where there was more than one additional household member, the proportion of others’ speech was computed by averaging the speech from all members. The model in R syntax was:

glm(PropOthersSpeaking~PropChildSpeaking * Community)

Unlike primary caregivers, other family members’ use of a language showed no systematic relationship with children’s language patterns (*OR* = 3.28, 95% CI [0.80, 14.45], *t* = 1.62, *p* = .104), nor did bilingual community predict language choices (*OR* = 0.94, 95% CI [0.22, 3.79], *t* = −0.09, *p* = .927). The interaction was also not significant (*OR* = 1.14, 95% CI [0.11, 12.33], *t* = 0.11, *p* = .912). These findings suggest that the amount that other household members spoke a language was not systematically related to how much the child spoke that language, regardless of bilingual community. The model showed limited predictive power (null deviance = 33.55, residual deviance = 31.08 on 52 df).

We additionally examined the reverse direction, whether other household members’ language use predicted children’s language use, using the following model shown in R syntax:

glm(PropChildSpeaking~PropOthersSpeaking * Community)

Children’s language use also showed no systematic relationship with other household members’ language patterns (*OR* = 3.03, 95% CI [0.81, 12.04], *t* = 1.62, *p* = .105), nor did community predict children’s language choices (*OR* = 0.92, 95% CI [0.24, 3.49], *t* = −0.12, *p* = .904). The interaction was also not significant (*OR* = 1.18, 95% CI [0.13, 11.07], *t* = 0.15, *p* = .884). Together, these bidirectional null results suggest that language use between children and other household members was not systematically coordinated in either direction. Note that as these models did not include individual predictors, both were run on the full Session B sample of 28 families (French–English: *n* = 16; Spanish–English: *n* = 12), including the two Spanish–English families excluded from Analysis 2 due to missing questionnaire data.

#### 3.4.4. Summary Language Use

These analyses reveal strong bidirectional matching between primary caregivers and children across both communities, with children’s language exposure and caregivers’ language ability influencing overall language use patterns. Other family members showed weaker coordination with children (and children did not align with other family members), highlighting the central role of primary caregivers in language alignment.

### 3.5. Analysis 3. Turn-by-Turn Language Alignment

To test for turn-by-turn alignment, we analyzed conversational turns from dyadic interactions of primary caregivers and their children across both sessions. For child responses to primary caregivers, we included 3267 turns from 18 French–English families (*M* = 97.89, *SD* = 76.78) and 1505 turns from 19 Spanish–English families (*M* = 79.21, *SD* = 60.66). For caregiver responses to children, we included 1779 turns from 19 French–English families (*M* = 93.63, *SD* = 77.27) and 1518 turns from 19 Spanish–English families (*M* = 79.89, *SD* = 60.51). For each turn, we created lagged variables capturing the previous speaker’s language choice and coded whether the current speaker matched (1) or switched (0) languages. Analyses were restricted to turns between the primary caregiver and target child; turns involving other speakers were excluded as the small and unequal number of families with each secondary speaker type present (fathers, siblings, grandparents) and the limited number of conversational turns between children and individual secondary caregivers precluded reliable estimates. Mixed-language utterances were also excluded, as these occur within a single speaker’s turn rather than across conversational exchanges. Regression models for child-to-caregiver responses were run on 35 families (French–English Session A: *n* = 18, Session B: *n* = 16; Spanish–English Session A: *n* = 17, Session B: *n* = 9), and caregiver-to-child responses on 36 families (French–English Session A: *n* = 19, Session B: *n* = 16; Spanish–English Session A: *n* = 17, Session B: *n* = 9), due to missing language exposure or language ability data and two French–English children producing no identifiable speech during turns in response to the primary caregiver.

#### 3.5.1. Overall Turn-by-Turn Alignment

We first examined overall patterns of language alignment across 38 sessions using multilevel logistic regression models with a logit link function. The dependent variable was language alignment, with session (Session A vs. Session B) and community (French–English bilinguals vs. Spanish–English bilinguals) as fixed effects, and random intercepts for families to account for individual variation in language preferences, see model in R syntax:

*glmer*(Alignment~Session * Community + (1|*Family*))

The results revealed a significant main effect of session (*OR* = 0.59, 95% CI [0.47, 0.74], *z* = −4.51, *p* < .001), indicating that the likelihood of language matching was lower in Session B than in Session A. The main effect of bilingual community was not significant (*OR* = 0.63, 95% CI [0.22, 1.81], *z* = −0.85, *p* = .393) and the session by community interaction was marginally significant (*OR* = 1.39, 95% CI [0.95, 2.03], *z* = 1.71, *p* = .087), suggesting numerically lower matching in Session B in the Spanish–English bilingual sample compared to the French–English sample. The model showed a good fit (AIC = 4974.7).

#### 3.5.2. Monte Carlo Simulations: Testing Turn-by-Turn Alignment Against Chance

The high matching rates observed could potentially reflect families’ individual language abilities (e.g., if both parent and child strongly prefer English, they will frequently “match” simply because both use English most of the time) rather than genuine turn-by-turn coordination. To test whether sequential dependencies between speakers exceed chance expectations, we conducted Monte Carlo simulations that compared observed matching rates against what would occur if speakers selected languages independently based on their own individual preferences.

This analysis required restricting the sample to sessions where chance-level matching would be meaningfully different from perfect matching. Specifically, we included only sessions that: (1) had somewhat balanced language use (<80% use of one language), as sessions where one primary language was used would show ceiling-level matching by chance alone, and (2) had sufficient conversational turns (≥30 turns) to generate stable baseline estimates. This resulted in a subsample of 30 sessions roughly evenly spread across Session A/Session B and communities (see Table 2 for a complete breakdown by community and session).

For each session meeting these criteria, we conducted Monte Carlo simulations (10,000 iterations per condition) that generated null distributions for each session. In each simulation, we randomly paired language choices while preserving each session’s observed frequency distributions of French, Spanish and English use. This approach allowed us to test whether sequential dependency between speakers’ language choices exceeded chance levels, while accounting for individual family differences in language preference.

**Figure 2:**
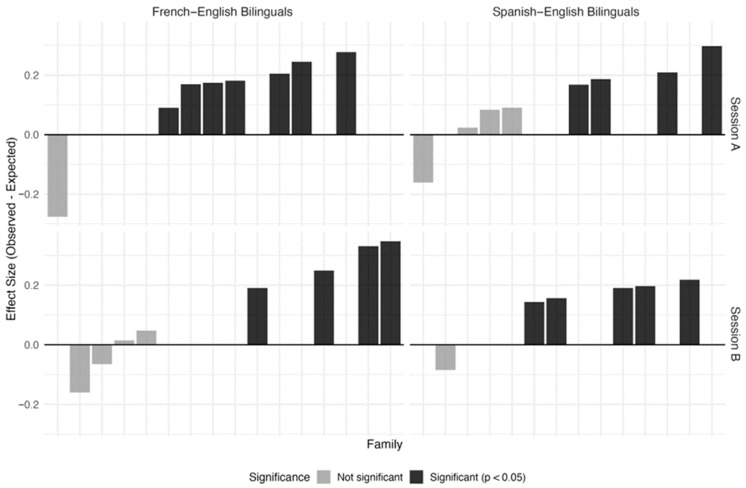
Effect sizes calculated using Cohen’s h comparing observed versus expected language matching rates for each family. Positive values indicate observed matching exceeded chance expectations based on Monte Carlo simulations. Black bars = significant alignment (*p* < .05); gray bars = non-significant. Families are separated by community and session (Session A = one-on-one; Session B = multi-party interactions).

Observed alignment rates substantially exceeded chance expectations for two-thirds of the analyzed sessions. Amongst the sessions exceeding chance, on both Session A and Session B, families’ observed alignment rate (Session A: *n* = 11, *Mobserved* = 0.77, *SD* = 0.05; Session B: *n* = 9, *Mobserved* = 0.75, *SD* = 0.06) was on average 20–22% higher than the expected rate (Session A: *Mexpected* = 0.57, *SD* = 0.06; Session B: *Mexpected* = 0.53, *SD* = 0.03). This represented a medium effect size (Session A Cohen’s *h* = 0.43; Session B Cohen’s *h* = 0.47), indicating bidirectional language adaptation beyond what would occur if speakers selected languages independently based solely on their individual language preferences. This pattern held across both bilingual communities (see Figure 2 for a graphical representation of these results).

One-third of the analyzed sessions did not show turn-by-turn alignment above their baseline, and for these sessions, we cannot reject the null hypothesis that observed matching reflects similar language preferences rather than moment-to-moment coordination. To better understand this pattern, we ran a set of exploratory analyses examining whether families showing non-significant alignment had different language balance profiles than those showing significant alignment (see Supplementary Materials). Our results showed that families where children received more balanced lifetime language input—that is, had more similar exposure to their two languages—showed smaller coordination effect sizes and were less likely to show statistically significant turn-by-turn alignment. We will return to this finding in Section 4. Finally, we cannot draw conclusions about the 37 sessions that could not be analyzed under this approach due to heavily unbalanced language use or too few conversational turns.

#### 3.5.3. Bidirectional Turn-by-Turn Alignment: Who Adapts to Whom?

To examine whether children and caregivers differ in their alignment patterns and what factors predict alignment strength, we fit separate multilevel logistic regression models for each speaker type. These models included session, community, and individual predictors as fixed effects, with random intercepts for families, and were run on all sessions for which dyadic turn data were available (*n* = 35 for child-to-caregiver and *n* = 36 for caregiver-to-child analyses), regardless of Monte Carlo significance, as the regression models address a distinct question from the chance-level comparisons above.

*Child Turn-by-Turn Alignment Model.* We examined children’s turn-by-turn alignment with primary caregivers using a generalized linear mixed model with a binomial distribution and logit link, including random intercepts for families. We first tested whether including child age as an additional predictor improved model fit. Model comparisons revealed that a model including session, community, child language exposure, and child age as interacting predictors significantly improved fit over a model without age, χ^2^(8) = 37.30, *p* < .001. Therefore, the final model was

*glmer*(Alignment~Session * Community * ChildLangExposure * ChildAge + (1|*Family*))

The model showed acceptable fit (AIC = 2445.5) with substantial between-family variance (random intercept variance = 1.40, *SD* = 1.18). Baseline matching in Session A for French–English families was high (*OR* = 10.54, 95% CI [5.00, 22.24], *z* = 6.18, *p* < .001, approximately 91% predicted match rate), with Spanish–English families showing comparable baseline matching (approximately 84% predicted match rate). Several higher-order interactions revealed the complexity of children’s language alignment patterns.

A significant three-way interaction between community, exposure, and age (*OR* = 1.67, 95% CI [1.27, 2.20], *z* = 3.66, *p* < .001) indicated that the relationship between language exposure and alignment, and how it changed with age, operated differently in the two communities. In French–English families, younger children showed a strong positive relationship between exposure and alignment that weakened as children grew older (*OR* = 0.57, 95% CI [0.46, 0.72], *z* = −4.88, *p* < .001). In Spanish–English families, the exposure-alignment relationship remained more consistent across ages, with a less pronounced age-related decline in the exposure effect.

A significant three-way interaction between session, community, and age (*OR* = 0.39, 95% CI [0.20, 0.75], *z* = −2.82, *p* = .005) revealed that older children showed larger decreases in caregiver alignment during Session B, which was particularly pronounced in Spanish–English families compared to French–English families. Overall, children were less likely to match caregivers in Session B (*OR* = 0.54, 95% CI [0.36, 0.80], *z* = −3.10, *p* = .002), with this decrease being particularly pronounced for older children in Spanish–English families. This could reflect either that older children align less to their primary caregivers as they develop more autonomous language choices, or that older children become more sensitive to the broader social context when multiple speakers are present, shifting their alignment toward other speakers—particularly in the Spanish–English community.

A significant three-way interaction between session, community, and exposure (*OR* = 2.15, 95% CI [1.06, 4.37], *z* = 2.11, *p* = .035) indicated that the role of language exposure in predicting alignment changed differently across sessions for the two communities. French–English families showed a strong positive effect of language exposure on alignment across both sessions (*OR* = 2.93, 95% CI [2.28, 3.78], *z* = 8.38, *p* < .001) with no significant change in Session B (*OR* = 0.81, 95% CI [0.54, 1.22], *z* = −1.01, *p* = .313). In Spanish–English families, the exposure effect was weaker overall (*OR* = 0.58, 95% CI [0.42, 0.79], *z* = −3.48, *p* < .001), but this community difference was attenuated in Session B, suggesting that multi-party contexts reduced the gap between communities in exposure-predicted alignment.

A significant three-way interaction between session, exposure, and age (*OR* = 1.61, 95% CI [1.10, 2.34], *z* = 2.46, *p* = .014) indicated that the combined influence of exposure and age on alignment shifted between sessions. The age-related pattern in the exposure effect described above—where younger children’s alignment was more strongly associated with language exposure—was attenuated in Session B. This suggests that developmental and input factors jointly modulate how children coordinate language choices across interactional contexts, with the joint influence of age and exposure on alignment being less pronounced in multi-party settings.

Community, age, and their two-way interaction were not significant main effects (all *p*s > 0.169), consistent with their effects being expressed through the higher-order interactions described above. The two-way interaction between session and age was marginal (*OR* = 1.50, 95% CI [0.96, 2.34], *z* = 1.78, *p* = .074), suggesting a trend toward greater session-related decreases in older children across both communities. The four-way interaction and all remaining two-way interactions were not significant (all *p*s > .155).

*Caregiver Turn-by-Turn Alignment Model.* We examined primary caregivers’ turn-by-turn alignment with children using a generalized linear mixed model with a binomial distribution and logit link, including random intercepts for families. The model included session, community and caregiver language ability as interacting predictors:

*glmer*(Alignment~Session * Community * CaregiverLangAbility + (1|*Family*))

The model showed acceptable fit (*AIC* = 2342.5) with substantial between-family variance (random intercept variance = 2.27, *SD* = 1.51). Baseline matching in Session A for French–English families was high (*OR* = 9.49, 95% CI [4.50, 20.03], *z* = 5.91, *p* < .001, approximately 90% predicted match rate), with Spanish–English families showing similarly high baseline matching (approximately 88% predicted match rate). In contrast to the complex pattern observed for children’s alignment, caregiver alignment showed a simpler structure. The only significant effect was session (*OR* = 0.64, 95% CI [0.45, 0.89], *z* = −2.64, *p* = .008), indicating that caregivers were less likely to match children in Session B compared to Session A. No significant effects emerged for community (*OR* = 0.74, 95% CI [0.25, 2.16], *z* = -0.55, *p* = .583), caregiver language ability (*OR* = 1.07, 95% CI [0.80, 1.43], *z* = 0.44, *p* = .660), or any of the interactions (all *p*s > 0.141), suggesting that caregivers’ turn-by-turn alignment with children was remarkably consistent across communities, caregiver proficiency levels, and sessions, with only the presence of additional household members reducing alignment strength.

**Figure 3:**
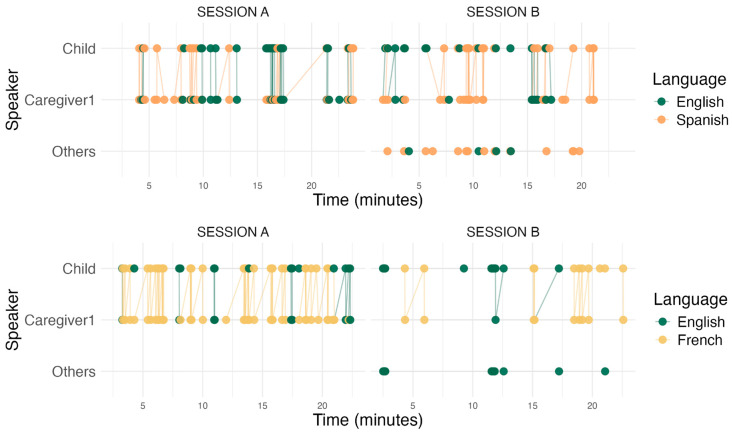
Examples of language use patterns from a family in each bilingual community. Each point represents a conversational turn, with color indicating the language used (top: green = English, orange = Spanish; bottom: green = English, gold = French). Diagonal lines connecting the Child and Caregiver rows indicate matched language turns between the two speakers. Rows show different speakers (Caregiver1 = primary caregiver present in both sessions; Child = target child; Others = additional household members present only in Session B, excluding the primary caregiver and the child). The x-axis shows time in minutes across the 20 min play sessions. Session A involved only the primary caregiver and child, while Session B included additional household members. The top panel shows a Spanish–English family; the bottom panel shows a French–English family. Visual inspection reveals moment-to-moment coordination between caregivers and children, with alignment more evident in dyadic contexts (Session A) than in multi-party contexts (Session B).

*Summary Turn-by-Turn Alignment.* Both children and caregivers showed high matching rates that decreased when other family members were present. In French–English families, the observed caregiver and child matching rates were 86% and 82% in Session A, and 79% and 75% in Session B, respectively. In Spanish–English families, the observed matching rates were 87% and 82% in Session A, and 73% and 68% in Session B, respectively. Both groups showed remarkably similar and consistently high alignment across sessions and communities (Figure 3 shows an example of turn-by-turn language alignment for a family from each community). Despite this overall similarity, caregivers’ alignment was less influenced by individual characteristics: while caregivers showed only a main effect of session, children’s alignment was modulated by language exposure and age through multiple interactions, varying by community. Children with greater language exposure showed stronger alignment with primary caregivers, though this effect was stronger for younger children and varied by community, and older children showed greater sensitivity to multi-party contexts, particularly in Spanish–English families. This asymmetry suggests that while both conversational partners coordinate their language choices at similarly high rates, children’s alignment is more dependent on developmental factors and language-specific experience, whereas caregivers maintain consistent responsiveness to children’s language choices regardless of their own proficiency or the social context.

## 4. Discussion

Bilingual children grow up in linguistically complex environments where multiple family members navigate decisions about which language to use, moment by moment. Drawing from the Systems Framework of Bilingual Development (Byers-Heinlein et al., 2025; Castellana & Benitez, 2026), this study investigated language alignment in 39 families with 18–35-month-old children, testing the degree to which caregivers and children match their language use during naturalistic interactions. We examined alignment across different interpersonal contexts (one-on-one versus multi-party interactions) and two bilingual communities with distinct sociolinguistic contexts: French–English families in Quebec, Canada, where both languages have official status, and Spanish–English families in New Jersey, United States, where Spanish functions as a heritage language with limited institutional support.

Our findings address three key research questions. First, we found that caregivers and children showed strong correspondence in their overall language choices. As predicted, this alignment was stronger between children and primary caregivers than between children and other household members, while the presence of additional household members reduced alignment between primary caregiver–child dyads. Second, contrary to our predictions, we found no significant differences in alignment patterns between the two bilingual communities, despite their markedly different sociolinguistic contexts. Third, individual factors including child age, language exposure, and caregiver language ability systematically predicted both overall language use and turn-by-turn alignment strength. Monte Carlo simulations confirmed that the observed matching tended to exceed what would occur if speakers selected languages independently based solely on their individual preferences, demonstrating genuine moment-to-moment coordination rather than merely reflecting aggregate similarity in language abilities. Below, we discuss each of these findings in turn.

### 4.1. Robust Alignment Between Caregivers and Children

The most striking pattern across our analyses was the robust correspondence between primary caregivers’ and children’s language choices, consistent with research showing the central contributions that mothers (who comprised most primary caregivers in our sample) can offer to bilingual development (Sander-Montant et al., 2025). Other family members, a group containing a large portion of fathers, showed numerically weaker and non-significant alignment with children’s language choices. Strong alignment with the primary caregiver alongside weaker alignment with other household members may be functionally important in bilingual families: if other family members consistently respond in a different language than the child primarily uses, they may provide exposure to the non-dominant language, creating a complementary distribution of language exposure within the family (De Houwer, 2011). This differs from primary caregivers’ code-switching, which reflects responsive adjustments to the child’s language, whereas other family members’ weaker alignment may stem from stable differences in language preference or dominance. Together, strongly and weakly aligned caregivers may play important but functionally distinct roles in supporting bilingual development, highlighting the unique contributions that fathers and other family members make to children’s language acquisition (Ferjan Ramírez et al., 2022).

Although matching remained high in absolute terms, both children and primary caregivers reduced their language alignment during multi-party interactions compared to one-on-one sessions (children: 82% → 73%; primary caregivers: 86% → 77%). This likely reflects the additional complexity of tracking multiple conversational partners simultaneously, managing turn-taking with more participants, and potentially navigating different language preferences from different family members (Genesee et al., 1996). However, reduced matching in multi-party contexts need not indicate a coordination failure: it could also reflect children’s emerging ability to flexibly accommodate different partners, necessarily reducing moment-to-moment matching with the primary caregiver. Consistent with this interpretation, neither children’s nor other household members’ language use systematically predicted the other’s language choices, suggesting that language coordination in multi-party contexts operates differently than in dyadic interactions and may involve more complex, less predictable dynamics (Comeau et al., 2003; Nicoladis & Genesee, 1996). We currently know little about what factors determine which language persists during group interactions, what triggers language switches, or which family members exert more influence over language choices. Future research examining longer stretches of multi-party conversation could illuminate the mechanisms underlying these collective coordination processes.

While these patterns demonstrate robust correspondence between caregivers’ and children’s language use, they raise a fundamental interpretive question: Does this correspondence reflect genuine moment-to-moment coordination, or does it simply indicate that family members share the same underlying language preferences or dominance patterns? This distinction has important theoretical implications. If correspondence merely reflects shared preferences, perhaps because both caregiver and child are English-dominant, then high matching rates could occur even without real-time adaptation between partners. In contrast, if correspondence reflects active coordination, then speakers are dynamically adjusting their language choices in response to each other’s behavior, demonstrating interpersonal synchrony.

We employed Monte Carlo simulations to address this question directly. The simulation results revealed important complexities. We could only analyze sessions that were somewhat balanced in their use of the two languages and produced sufficient turns in both languages. Among analyzable sessions, observed matching exceeded simulated baselines significantly above chance in 67% of sessions, indicating genuine turn-by-turn coordination, while in the remainder of sessions, matching was not statistically different from chance.

The variability that we observed amongst sessions could indicate that different families and/or individuals employ different coordination strategies. Some may prioritize linguistic consistency within conversations, actively maintaining whichever language is in use and adjusting when their partner switches. Others may operate more independently, with each speaker using their preferred language based on comfort or goals rather than continuously adapting turn-by-turn. Alternatively, families might coordinate primarily at the conversation level—establishing which language to use early in an interaction and then maintaining that choice (Lanza, 1997)—rather than engaging in continuous moment-to-moment adaptation. Exploratory analyses suggested that children with more balanced language input experienced less moment-to-moment matching. A possible explanation is that families that use both languages more interchangeably across caregiving contexts may rely on more fluid discourse strategies beyond sequential turn-by-turn matching. Children in these families may also be more flexible and proficient users of both languages, reducing the need for explicit turn-by-turn convergence. This interpretation is consistent with findings that more balanced language input is associated with greater language mixing (Kremin et al., 2022).

Importantly, the distinction between “shared preferences” and “active coordination” may be less stark than it initially appears. Shared preferences in bilingual families do not emerge randomly—they develop through repeated interactions where family members influence each other over time (De Houwer & Bornstein, 2016; Lanza, 1997), gradually converging on patterns that facilitate communication. From this developmental systems perspective, shared preferences are themselves a product of ongoing coordination processes, operating across different timescales: stable language preferences reflect an accumulated history of interaction, while moment-to-moment coordination reflects real-time responsiveness to a partner’s language choices. Rather than competing explanations, these represent complementary levels of analysis, and the convergence of evidence across our metrics suggests that bilingual families coordinate their language use both at moment-to-moment and across longer developmental timescales.

### 4.2. Community Context Moderates Individual Predictors of Language Alignment

We predicted that French–English families in Quebec would show different alignment patterns than Spanish–English families in New Jersey, reflecting contrasting sociolinguistic contexts. This was based on the Systems Framework of Bilingual Development (Byers-Heinlein et al., 2025), whereby we expected societal differences to cascade down, influencing interpersonal-level coordination between caregivers and children. Overall, our results suggest that interpersonal dynamics within the family shape the fundamental coordination process, while broader societal factors modulate how individual characteristics predict alignment strength during this early period. Primary caregivers in both communities showed similar baseline alignment with children, and this coordination was unaffected by community context. Primary caregivers seem to create relatively stable language-learning environments that operate according to similar interpersonal principles, which could include maintaining connection, facilitating comprehension, and supporting communication (Rowe & Snow, 2020) regardless of the broader sociolinguistic context.

However, community context *did* shape how individual characteristics influenced children’s alignment. Children’s language exposure predicted alignment in both communities, but this effect was significantly weaker in Spanish–English families. Additionally, the relationship between exposure and alignment changed with age differently across communities: younger French–English children showed a strong positive relationship between exposure and alignment that weakened as they grew older, whereas in Spanish–English families, the age-related decline in the exposure-alignment relationship was less pronounced. These community-specific patterns likely reflect the different language ecologies children experience. Montreal’s strong institutional support for French—including official bilingualism and French schooling—may provide French–English children with rich language exposure across multiple contexts, reducing their dependence on within-family exposure patterns as they develop. These community-specific patterns may reflect differences in family language policies across the two contexts. Quebec’s strong institutional bilingualism may foster ideologies that support both languages equally, reducing children’s dependence on within-family exposure, whereas families in the more English-dominant New Jersey context may adopt more deliberate heritage language management strategies (Curdt-Christiansen, 2009) that tie children’s alignment more closely to family-specific input patterns. In contrast, Spanish–English children in a more English-dominant environment may continue to rely heavily on family-specific language exposure throughout early childhood.

Community context also influenced language choice patterns when additional household members were present. During multi-party interactions, French–English children decreased their English use (from 49% to 38%), while Spanish–English children increased theirs (from 55% to 61%), potentially reflecting different community-level language maintenance dynamics and extended family language profiles. Primary caregiver proficiency patterns provide insight into these dynamics. French–English caregivers showed relatively balanced bilingual proficiency (English *M* = 6.85, French *M* = 6.30) with consistent French skills across families (*SD* = 1.17, range = 3–7). In contrast, Spanish–English caregivers, while showing similar English proficiency (*M* = 6.65), reported lower Spanish proficiency (*M* = 5.94) with greater variability (*SD* = 1.75, range = 1–7), suggesting these families span a continuum from strong intergenerational Spanish transmission to a potential language shift toward English. These proficiency differences likely extend to other household members such as older siblings (Bridges & Hoff, 2014), shaping the broader household language dynamics in community-specific ways.

This pattern reveals how different system levels interact during early childhood within the Systems Framework. While development emerges from interactions across individual, interpersonal, and societal levels, the strong moment-to-moment alignment we observed between primary caregivers and children operated through consistent interpersonal mechanisms across communities, even as societal factors shaped the developmental pathways leading to successful coordination. Family interactions create stable language-learning contexts where the fundamental coordination process remains similar regardless of broader sociolinguistic pressures, though community context influences which individual characteristics facilitate this coordination and how they change with development (e.g., see example of stability in interpersonal dynamics despite societal variation from non-English language use during COVID lockdowns in the US, Bulgarelli & Potter, 2024).

Community effects may also operate through additional mechanisms we did not measure. Beyond moderating how individual characteristics predict alignment, the ways families achieve coordination may differ across communities in ways not captured by matching rates alone. For example, community context could affect the specific strategies employed or meanings attached to language choices without affecting observable matching rates. French–English families might maintain alignment through consistent language use within conversations, while Spanish–English families might achieve similar alignment through flexible accommodation after switches. Future research examining fine-grained conversational patterns could reveal whether these qualitative differences in coordination strategies exist across communities.

Finally, community effects may emerge more strongly at later developmental stages. Our study focused on preschool children, and as their social worlds expand to include peers, educators, and media representing the broader community, societal factors may shape language preferences and behaviors in ways that feed back into family interactions more directly. The community effects we observed during toddlerhood—operating primarily through moderation of how exposure and age predict alignment rather than through direct effects on alignment strength itself—may become more pronounced as children develop. For instance, community context might begin to directly influence alignment strength rather than only shaping the pathways through which children achieve coordination. Longitudinal research tracking families across this transition would clarify whether and how the relative influence of interpersonal versus societal factors shifts as children’s linguistic and social worlds expand beyond the family context.

### 4.3. Predictors of Language Alignment

Several individual characteristics systematically shaped coordination dynamics, with effects that sometimes varied by community context as described above.

Children demonstrated stronger alignment when they had greater proficiency in a language, as measured by their relative exposure to each language. This finding extends well-established links between exposure and vocabulary development in bilingual children (Byers-Heinlein et al., 2024; Place & Hoff, 2011) to show that accumulated experience also shapes real-time coordination. As noted above, this exposure effect was weaker in Spanish–English families, and the relationship between exposure and alignment changed differently with age across the two communities: younger French–English children showed a strong positive relationship between exposure and alignment that weakened as they grew older, whereas Spanish–English children showed more consistent exposure-alignment relationships across ages.

Child age showed complex, context-dependent effects. While older children did not show stronger baseline alignment overall, the benefits of exposure for alignment were stronger among younger children. This suggests that as children develop broader language skills, they may become less dependent on language-specific exposure for successful coordination (Yurovsky et al., 2016). Additionally, older children’s alignment was more strongly disrupted by the presence of multiple speakers than younger children’s, particularly in Spanish–English families. This may reflect older children’s greater ability to flexibly adjust their language choices across conversational partners (Sheng et al., 2013, Gross et al., 2022), or their heightened sensitivity to implicit family norms about language use with specific individuals (e.g., understanding that Spanish is typically spoken with grandparents while English is used with older siblings; Tare & Gelman, 2010; Genesee et al., 1996). More broadly, these age-related changes suggest a developmental progression in children’s ability to track language and speaker identity simultaneously. While infants show limited ability to associate speakers with languages in controlled laboratory tasks (Schott et al., 2023; Potter & Lew-Williams, 2025), toddlers with accumulated naturalistic experience appear to develop more sophisticated coordination abilities. The older children in our sample showed greater sensitivity to multi-party contexts while being less dependent on language-specific exposure for alignment, suggesting they increasingly integrate speaker identity, language choice, and social context in their real-time language decisions. This development likely reflects the combined influence of cognitive maturation and repeated interactions with responsive caregivers who model consistent alignment patterns (Gross et al., 2022; Byers-Heinlein et al., 2025). It is important to note that younger children produced more unidentifiable speech in our data, which likely reduced our statistical power to detect robust alignment at younger ages, particularly during multi-party interactions when children’s overall speech was reduced.

In contrast to the complex pattern observed for children, caregiver alignment showed remarkable simplicity and consistency. Caregiver language proficiency did not predict how closely caregivers matched their children turn-by-turn, and caregiver alignment was unaffected by community, session context, or any interactions among these factors. The only significant effect for caregivers was session, with all caregivers showing reduced alignment in multi-party contexts regardless of their proficiency levels or community context. This dissociation between child and caregiver patterns suggests that caregivers maintain consistent responsiveness to children’s language choices independently of their own linguistic abilities—a pattern that may reflect the relatively high bilingual proficiency of caregivers in our sample, as previous work has shown that caregiver language dominance can influence the degree of alignment to their children (Slawny et al., 2025).

### 4.4. Limitations and Future Directions

This work has some limitations. First, while our Monte Carlo simulation approach could only be applied to sessions with relatively balanced language use and sufficient conversational turns, reducing our statistical power to detect individual differences in alignment patterns. Longer interaction sessions or full-day home recordings would allow this approach to be applied more broadly. Second, coders could not always identify the language speakers were using during interactions, particularly for younger children with limited verbal production. Interestingly, it is also possible that caregivers cannot always classify a child’s language use, which could make it harder for them to align, especially with younger children and in multi-party contexts.

Third, because multiple speakers were always present during Session B, we captured fewer conversational turns between children and each secondary caregiver, limiting our conclusions about alignment patterns with specific other family members, despite their important contributions to bilingual development (Ferjan Ramírez et al., 2022). Future work with dedicated one-on-one sessions with multiple family members and larger samples would allow for more fine-grained conclusions about how children coordinate their language choices with different members of their social network. Fourth, our study focused on observed language practices rather than other aspects of bilingual interaction such as pragmatic functions of code-switching or family language ideologies. Combining quantitative alignment measures with assessments of family language policy and ideologies (Spolsky, 2012; K. A. King & Fogle, 2013) would provide a more complete picture of how community context shapes bilingual interaction. Families with similar alignment rates may hold very different beliefs about language mixing or employ different strategies for promoting their two languages.

Fifth, there are several considerations regarding the representativeness and power of our sample. Dropout between sessions was unequally distributed across communities, with more Spanish–English families failing to complete Session B and missing questionnaire data also concentrated in this group, which might have made some analyses underpowered, explaining some of our marginal interactions. Future work with larger samples would allow for more robust conclusions about alignment patterns in multi-party contexts. Additionally, both samples were highly educated, with over 70% of caregivers holding a bachelor’s degree or above. The highly educated nature of our sample may limit generalizability to broader bilingual populations, and future work should examine whether alignment patterns replicate across more socioeconomically diverse samples.

Sixth, our snapshot of one developmental period (18–35 months) cannot capture how alignment evolves as children’s social world expands into preschool and formal education, when societal-level influences cascade more directly into family language practices. Longitudinal research tracking these transitions would clarify whether the community-level moderation we observed strengthens over development.

Finally, the present study focuses on turn-by-turn language matching but does not consider the frequency or directionality of code-switching. This is an important dimension given that children must acquire not only community-level language preferences but also community-specific code-switching patterns from their caregivers, which vary not only across but within communities (Poplack, 1988). Families with higher switching rates may show different alignment dynamics, and future work should examine how code-switching relates to alignment strength and how it interacts with child age. Preliminary evidence from the same dataset indicates that primary caregivers have relatively stable code-switching behavior across sessions, yet there was considerable variability across families and communities in switching rates and types (Kosie et al., 2026). Given these complex interactions, how code-switching frequency and alignment interact across development and contexts remains an important open question.

### 4.5. Implications for Theory, Practice, and Policy

Our findings have implications across multiple levels. For bilingual families, our findings offer reassurance: regardless of the broader linguistic environment, strong alignment with primary caregivers appears to be a natural feature of early bilingual interaction across diverse contexts. Families need not have perfectly balanced bilingualism to achieve this coordination, as alignment emerged even among caregivers with varying proficiency levels. At the same time, the reduced alignment when multiple caregivers were present suggests that other family members influence children’s language choices, highlighting the distributed nature of language input and the importance of considering multiple caregivers in supporting bilingual development.

Beyond practical implications, this work carries methodological lessons for bilingual acquisition research more broadly. Our finding that community context modulated the role of individual predictors—particularly child age and language exposure—suggests that findings from single-community studies may not generalize across bilingual contexts. A pattern that holds in one sociolinguistic environment may look quite different in another, or may not emerge at all. This underscores the importance of comparative cross-community designs to help disentangle general features of bilingual development from those that are context-specific.

From a policy perspective, these findings underscore the critical importance of quality time between children and their caregivers during early development. The strong alignment we observed suggests that caregivers who follow their children’s momentary language choices create opportunities for children to actively engage with objects, events, and people in their preferred language, potentially reinforcing language learning in specific contexts. At the same time, the slight reduction in alignment during multi-party contexts indicates that exposure to multiple family members may support bilingual development in complementary ways, perhaps by exposing children to diverse language models and conversational dynamics. This evidence supports policies that extend parental leave programs to enable not only primary caregivers but also second parents and other family members to spend substantial time with young children (Fibla et al., 2022). Such policies would acknowledge the distributed nature of early language learning and support the rich interpersonal interactions—both dyadic and multi-party—that foster bilingual acquisition.

## 5. Conclusions

This study reveals that language alignment between caregivers and young bilingual children operates through similar interpersonal coordination processes across diverse sociolinguistic contexts. At the same time, community context shaped how individual characteristics influenced this coordination, particularly the roles of language exposure and age. These findings bridge the family language policy tradition, which has emphasized parental ideologies and reported practices, with the language acquisition tradition focused on input quantity and quality, by capturing the enacted dimension of family language policy during real-time interaction. From a policy perspective, the robust alignment we observed in dyadic interactions, combined with the complementary role of other household members, underscores the importance of policies that enable not only primary caregivers but also second parents and extended family members to spend substantial time with young children, supporting the distributed interpersonal interactions that foster bilingual acquisition.

## Figures and Tables

**Table 1:** Mean percentage of English use by session, bilingual community, and speaker.

Community	Session	Speaker	English Use M (SD)	Range
French–English	Session A	Children	49.42% (41.13)	0–100%
French–English	Session A	Primary Caregiver	53.13% (41.11)	0–100%
French–English	Session B	Children	38.16% (34.93)	0–100%
French–English	Session B	Primary Caregiver	51.35% (36.97)	0–99%
French–English	Session B	Other Family Members	39.98% (36.78)	1–98%
Spanish–English	Session A	Children	55.36% (35.59)	0–100%
Spanish–English	Session A	Primary Caregiver	54.97% (36.08)	3–100%
Spanish–English	Session B	Children	60.80% (32.11)	1–100%
Spanish–English	Session B	Primary Caregiver	48.61% (34.32)	12–99%
Spanish–English	Session B	Other Family Members	45.26% (34.18)	3–99%

Note. Session A = single caregiver present; Session B = multiple household members present. Children = the target child of the study. Primary Caregiver = the caregiver present in both sessions. Other Family Members = additional household members present in Session B only, excluding the target child and the primary caregiver from Session A. Values represent the mean percentage of English speech out of total identifiable speech averaged across families. Standard deviations are shown in parentheses. Range indicates the minimum and maximum percentages observed.

**Table 2:** Sample attrition and alignment results from the Monte Carlo simulations.

Community	Session	Initial Sample	Excluded > 80%	Excluded < 30	Included	Sig.	NS
French–English	A	20	12	0	8	7	1
French–English	B	16	6	2	8	4	4
Spanish–English	A	19	10	1	8	4	4
Spanish–English	B	12	3	3	6	5	1
TOTAL		67	31	6	30	20	10

Note. Session A = single caregiver present; Session B = multiple household members present. Initial sample = number of families recorded. Excluded > 80% = families excluded because one speaker used a single language more than 80% of the time, preventing meaningful analysis of language coordination. Excluded < 30 turns = families excluded due to having fewer than 30 conversational turns. Note that 7 families were excluded due to both reasons. Included = final sample size for alignment analyses. Significant = *n* families showing significant language alignment (*p* < .05). NS = *n* families showing non-significant alignment.

## Data Availability

Study materials, anonymized data, and analysis scripts are publicly available in this OSF project Fibla et al. (2026, April 1).

## Supplementary Materials

The following supporting information can be downloaded at https://www.mdpi.com/article/10.3390/bs16050788/s1: Figure S1: Language balance predicts chance-level matching rate. Greater exposure to the lesser-heard language (more balanced input) is associated with lower baseline chance rates. Points show individual families, color coded by bilingual community; Figure S2: Language balance and coordination effect size. More balanced families (higher lesser-heard exposure) show smaller coordination effect sizes, suggesting shared language dominance facilitates detectable alignment. The dashed horizontal line indicates an effect size of zero (observed matching rate equal to chance baseline). Point shape indicates whether alignment was statistically significant per each individual family; Figure S3: Language balance and probability of significant alignment. More balanced families (higher lesser-heard language exposure) tend to show higher Monte Carlo *p*-values, indicating that shared language dominance facilitates statistically detectable coordination. The dashed line marks *p* = .05. Point shape indicates whether alignment was statistically significant; Table S1: Lesser-Heard Language Exposure (Proportion) by Community and Session; Table S2: Language Balance by Monte Carlo Significance: non-significant sessions had more balanced, not less balanced, input.
