# Supplementary material for: Bilingual Families Align Their Languages During Naturalistic Interactions: Evidence from Two Bilingual Communities
Source: Behav Sci (Basel). 2026 May 15;16(5):788. doi: 10.3390/bs16050788 (PMC13203572; doi:10.3390/bs16050788)
Supplement: Supplementary file 1 [file behavsci-16-00788-s001.zip › behavsci-4264885-supplementary.pdf]

Supplementary Materials: Language Balance Effects on Turn-by-Turn Alignment

The main Monte Carlo analysis reported that 1/3 of the analyzed sessions did not show turn-by-turn alignment above their chance baseline. This raises the question of whether families' specific language profiles, such as the degree of language balance within the included sample, explains who shows significant alignment and who does not even after passing the 80% exclusion threshold.

The analyses reported in this supplementary material address this question by examining whether language balance — operationalised as children's reported lifetime exposure to their lesser-heard language (0% = monolingual, 50% = fully balanced) — predicts the Monte Carlo alignment results. This measure is drawn from the parental report questionnaire described in the main manuscript. It reflects the percentage of time children are exposed to each language across their daily caregiving interactions, and is used here as a proxy for overall family language balance. The analyses reported below are exploratory and we did not have a predicted pattern.

Three supplementary analyses are reported below:

1. **Supplementary Analysis 1:** Does language balance predict chance-level matching rates?
2. **Supplementary Analysis 2:** Does language balance predict coordination effect sizes?
3. **Supplementary Analysis 3:** Does language balance predict the probability of reaching statistical significance?

Descriptive Statistics

Table S1. Lesser-Heard Language Exposure (Proportion) by Community and Session.

Community	Session	M	SD	Min	Max	n
French-English	A	39.0	8.6	25.0	50	8
French-English	B	40.2	9.3	25.0	50	8
Spanish-English	A	39.2	7.7	29.5	50	8
Spanish-English	B	37.7	8.9	24.5	49	5

Table S2. Language Balance by Monte Carlo Significance: non-significant sessions had more balanced, not less balanced, input.

Alignment	n	M	SD
Non-significant	10	45.2	5.8
Significant ($p < .05$)	19	36.0	7.6

Supplementary Analysis 1: Language Balance and Chance-Level Matching

In the Monte Carlo simulation, each family-session has an expected matching rate — the rate of language matching that would occur by chance alone if speakers chose languages independently based solely on their own individual language preferences, with no turn-by-turn coordination. Families with very unbalanced input (e.g. one language used 75% of the time) tend to have higher chance baselines simply because both speakers independently gravitate toward the same dominant language. This analysis checks whether language balance predicts this chance baseline within the included sample, as a methodological validation: if it does not, then the findings in Supplementary Analyses 2 and 3 cannot be explained as artefacts of how chance baselines are calculated for families with different language profiles.

We examined whether language balance predicted chance-level matching rates using a linear model controlling for session and community [*lm(ExpectedMatchRate ~ LesserHeardLanguage + Session + Community)*]. Language balance was not a significant predictor of chance-level matching rates (b = 0.001, *95% CI* [-0.002, 0.003], t = 0.47, p = .642). No other effects were significant (all $ps > .356$), indicating that within the included sample, neither language balance, session, nor community reliably predicted how high the chance baseline was. This suggests that the 80% exclusion criterion applied in the main analysis was sufficient to remove the most extreme cases where chance-level matching would be inflated, and variation in language balance within the included sample does not distort the baseline. The findings in Supplementary Analyses 2 and 3 can therefore be interpreted as reflecting genuine differences in coordination rather than differences in chance-level matching.

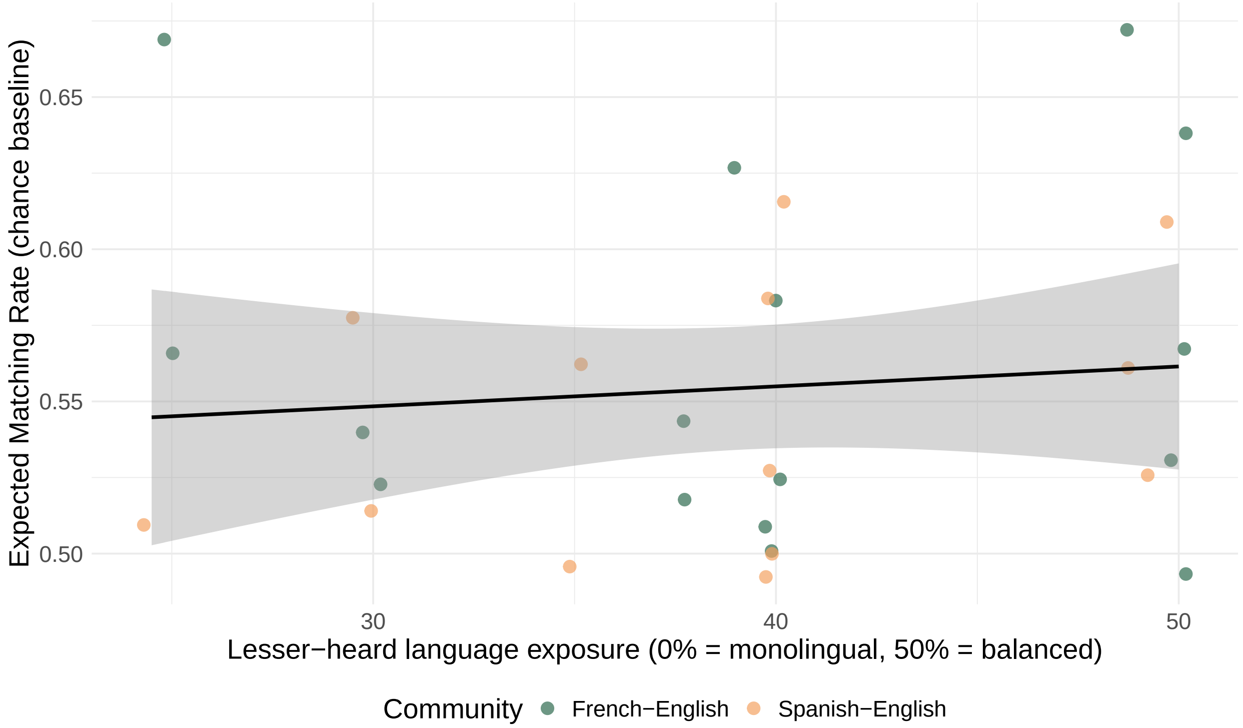

Figure S1. Language balance predicts chance-level matching rate. Greater exposure to the lesser-heard language (more balanced input) is associated with lower baseline chance rates. Points show individual families, color coded by bilingual community

Supplementary Analysis 2: Language Balance and Coordination Effect Size

We examined whether language balance predicted coordination effect sizes (computed as the difference between observed and expected matching rates) using a linear model controlling for session and community [*lm(Difference ~ LesserHeardLanguage + Session + Community)*]. In this context, a positive effect size would predict coordination above baseline. An effect size of 0 would indicate that a family matched exactly as often as chance would predict and there would be no evidence of coordination beyond language preferences. A negative effect size would indicate that a family matched less often than chance would predict, perhaps driven by increased language switching. Language balance was a significant negative predictor of coordination effect size (b = -0.008, *95% CI* [-0.015, -0.001], t = -2.49, p = .020), indicating that more balanced families showed smaller coordination effects above their chance baseline. No other effects were significant (all ps > .721). This suggests that families with stronger shared language dominance coordinate more consistently around their dominant language, producing larger and more detectable alignment effects above chance.

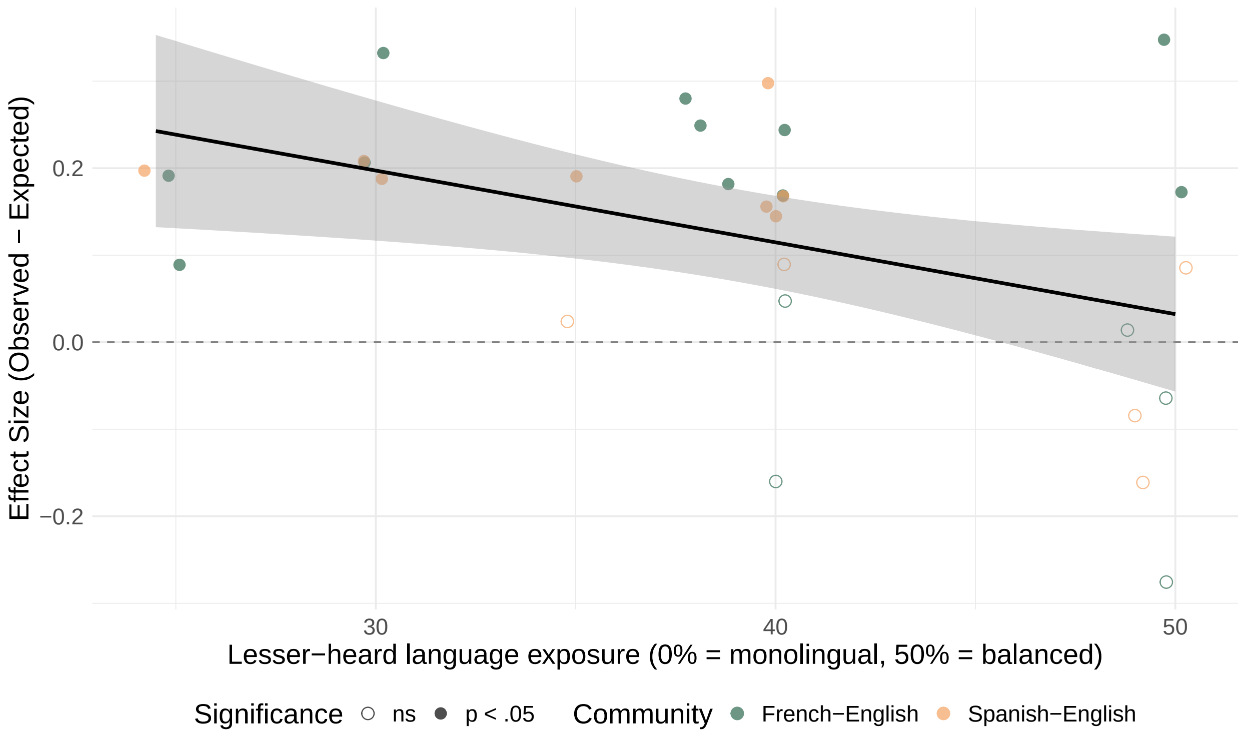

Figure S2. Language balance and coordination effect size. More balanced families (higher lesser-heard exposure) show smaller coordination effect sizes, suggesting shared language dominance facilitates detectable alignment. The dashed horizontal line indicates an effect size of zero (observed matching rate equal to chance baseline). Point shape indicates whether alignment was statistically significant per each individual family.

Supplementary Analysis 3: Language Balance and Probability of Significant Alignment

Supplementary Analyses 1 and 2 examine balance as a continuous predictor of alignment strength. Here, we examine whether balance also predicts whether a session crosses the significance threshold, combining a group comparison (t-test) with a logistic regression that controls for session and community.

Sessions showing significant alignment in the Monte Carlo test had less balanced language input (M = 36%, SD = 7.6%) compared to non-significant sessions (M = 45.2%, SD = 5.8%), and this difference was significant ($t(23.23) = 3.66$, $p = .001$). A logistic regression controlling for session and community [*glm(SignificantlyAligned ~ LesserHeardLanguage + Session + Community)*] confirmed this pattern. Exposure to the lesser-heard language was a significant negative predictor of alignment significance ($OR = 0.81$, *95% CI* [0.67, 0.93], z = -2.55, $p = .011$), indicating that families with less balanced input were more likely to show statistically significant alignment. No other significant effects emerged (all ps > .390).

Taken together with Supplementary Analysis 2, these findings suggest that shared language dominance is associated with turn-by-turn coordination.

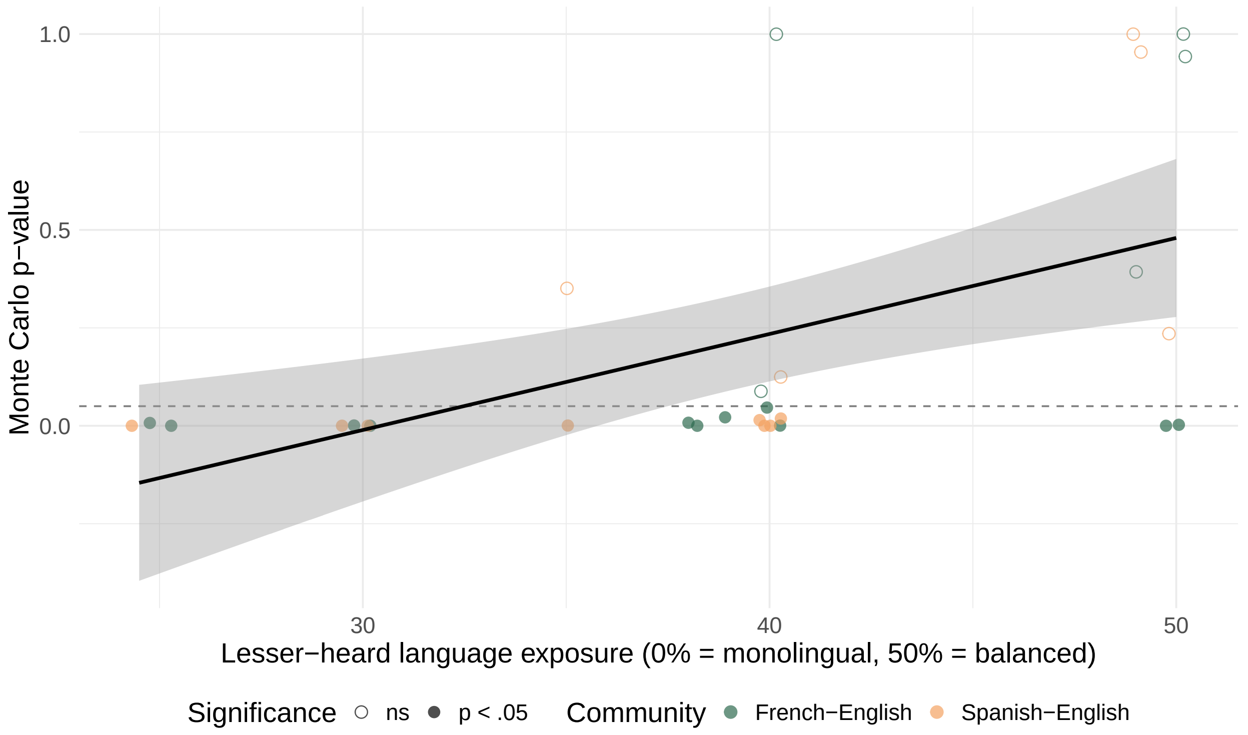

Figure S3. Language balance and probability of significant alignment. More balanced families (higher lesser-heard language exposure) tend to show higher Monte Carlo p-values, indicating that shared language dominance facilitates statistically detectable coordination. The dashed line marks p = .05. Point shape indicates whether alignment was statistically significant.

Overall Summary

Across the three supplementary analyses, a consistent pattern emerged: families where children received more balanced lifetime language input — that is, those with similar exposure to their two languages — showed smaller coordination effect sizes and were less likely to show statistically significant alignment. Language balance did not predict the chance-level baseline, ruling out the possibility that these effects are driven by ceiling-level chance matching.

These findings directly address the question of whether the non-significant one-third of sessions simply reflected more imbalanced language use: they did not. Non-significant sessions were in fact *more* balanced than significant ones. This pattern suggests that the 80% exclusion threshold used in the main Monte Carlo analysis was appropriate, and that within the included sample, variation in language balance reflects a genuine dimension along which coordination operates, rather than a methodological confound.
